# Low-dose omega-3 fatty acids in margarines prove disappointing as secondary prevention

**Published:** 2010

Lower doses of ω-3 fatty acids, given in the form of enriched margarines, did not reduce the overall rate of major cardiovascular events following myocardial infarction, according to results from the multicentre Alpha Omega trial. However, there were borderline significant reductions among post-MI sub-groups, including diabetic patients and women receiving alpha-linolenic acid (ALA) supplements, said principal investigator Daan Kromhout from Wageningen University, the Netherlands, in a hot-line session at the ESC.

The primary endpoint of the Alpha Omega trial was major cardiovascular events (MACE) including revascularisation by PCI or CABG. Important secondary endpoints included fatal coronary heart disease and ventricular arrhythmiarelated events, defined as sudden death, cardiac arrest and ICD placement.

A total of 4 837 patients (78.2% men, 21.8% women) aged between 60 and 80 years were enrolled in the double-blind trial. All had suffered an MI approximately four years before the placebo-controlled study began and were on medication which included antithrombotic agents (98%), antihypertensives (90%) and lipidlowering drugs (86%).

They were randomly assigned to daily use of one of four trial margarines for 40 months. The first was a placebo and the other three were supplemented with various combinations of ALA, eicosapentenoic acid (EPA), and docosahexaenoic acid (DHA). The combinations were as follows: ALA (2 g/day); EPA + DHA (400 mg/day); and a margarine containing EPA + DHA (400 mg/day) + ALA (2 g/day). All margarines were similar in taste and appearance for the four treatment groups. These margarines were used on bread instead of regular margarine or butter.

Results from the study, which was simultaneously published in the New England Journal of Medicine, showed that 671 patients (13.9%) suffered a major cardiovascular event during the follow-up period. This primary endpoint was not reduced by ALA (p = 0.02) or EPA + DHA (p = 0.93). This trial, which was carried out in collaboration with 32 hospitals in the Netherlands, did show a reduction in the rate of cardiovascular events, approaching significance in the sub-group of women receiving ALA supplements (p = 0.07).

A post hoc exploratory analysis of data from diabetic patients in the trial showed reductions in cardiovascular endpoints with EPA + DHA when compared with placebo. The strongest effects – reductions of approximately 50% – were on rates of fatal CHD and arrhythmia-related events in these diabetic patients.

